# C57Bl/6 N mice on a western diet display reduced intestinal and hepatic cholesterol levels despite a plasma hypercholesterolemia

**DOI:** 10.1186/1471-2164-13-84

**Published:** 2012-03-06

**Authors:** Charles Desmarchelier, Christoph Dahlhoff, Sylvia Keller, Manuela Sailer, Gerhard Jahreis, Hannelore Daniel

**Affiliations:** 1 Molecular Nutrition Unit, Technische Universität München, Molecular Nutrition Unit, Gregor-Mendel-Strasse 2, 85350 Freising Weihenstephan, Germany; 2 PhD Graduate School 'Epigenetics, Imprinting and Nutrition', ZIEL -Research Center for Nutrition and Food Sciences, Technische Universität München (TUM), 85350 Freising Weihenstephan, Germany; 3 Friedrich Schiller University, Institute of Nutrition, 07743 Jena, Germany

## Abstract

**Background:**

Small intestine and liver greatly contribute to whole body lipid, cholesterol and phospholipid metabolism but to which extent cholesterol and phospholipid handling in these tissues is affected by high fat Western-style obesogenic diets remains to be determined.

**Methods:**

We therefore measured cholesterol and phospholipid concentration in intestine and liver and quantified fecal neutral sterol and bile acid excretion in C57Bl/6 N mice fed for 12 weeks either a cholesterol-free high carbohydrate control diet or a high fat Western diet containing 0.03% (w/w) cholesterol. To identify the underlying mechanisms of dietary adaptations in intestine and liver, changes in gene expression were assessed by microarray and qPCR profiling, respectively.

**Results:**

Mice on Western diet showed increased plasma cholesterol levels, associated with the higher dietary cholesterol supply, yet, significantly reduced cholesterol levels were found in intestine and liver. Transcript profiling revealed evidence that expression of numerous genes involved in cholesterol synthesis and uptake via LDL, but also in phospholipid metabolism, underwent compensatory regulations in both tissues. Alterations in glycerophospholipid metabolism were confirmed at the metabolite level by phospolipid profiling via mass spectrometry.

**Conclusions:**

Our findings suggest that intestine and liver react to a high dietary fat intake by an activation of *de novo *cholesterol synthesis and other cholesterol-saving mechanisms, as well as with major changes in phospholipid metabolism, to accommodate to the fat load.

## Background

Obesity is an underlying risk factor in the development of cardiovascular diseases and is frequently associated with hypercholesterolemia and dyslipidemia [1-3]. Dyslipidemia is characterized by elevated plasma levels of triacylglycerides (TG), very low-density lipoprotein (VLDL), low-density lipoprotein (LDL), total cholesterol and decreased levels of high-density lipoprotein (HDL) [4].

Whereas liver, endothelium and adipose tissue have been extensively studied in the context of hypercholesterolemia, dyslipidemia and cardiovascular diseases, the small intestine has long been neglected. A high dietary intake of fat via a Western-style diet requires the epithelium of the upper small intestine to digest and absorb large quantities of dietary TG, sterols and phospholipids (PL) [5]. Uptake of lipid constituents such as free fatty acids and monoacylglycerols is carried out by transport proteins like the fatty acid transporter FAT/CD36 [6], possibly the fatty acid transport protein 4 (FATP-4) [7,8] and in addition via fatty acid flip-flop mechanisms. TG are then re-synthesized in enterocytes and assembled into chylomicrons (CM) which, together with other lipophilic compounds, including the sterols, are released via lymph vessels into the blood circulation [9]. Uptake of dietary cholesterol into epithelial cells involves the Niemann-Pick C1 Like Protein 1 (NPC1L1), the target of the cholesterol-lowering drug ezetimibe [10-12], and possibly the scavenger receptor class B1 (SR-B1) and CD36 [13]. Cholesterol, like other dietary sterols, can also be exported back from the enterocyte into the lumen by the ATP-binding cassette sub-family G member 5 and 8 proteins (ABCG5 and -8) [14]. However, cholesterol is also synthesized *de novo *in epithelial cells [15] and then exported together with TG via CM. Bile acids, released from the gallbladder after meal intake, are mainly absorbed in the terminal ileum via a specialized Na^+^-dependent transporter [16], while PL, released together with bile acids, may undergo complete hydrolysis in more proximal regions and follow the absorption of the other dietary lipids [17].

As hypercaloric diets usually provide large quantities of fat [18], the intestine is forced to adapt to the lipid overload by increasing its absorption capacity [19] through an increase in its absorptive surface area and an upregulation of genes encoding for proteins involved in lipid uptake and processing [20,21]. The capacity for postprandial intestinal lipoprotein secretion has been found to be increased upon high fat intake [22] and this effect was observed as early as after 7 days of feeding [23]. The rise in circulating CM is thought to contribute to atherogenesis and is considered as a risk factor for cardiovascular diseases, emphasizing the prominent role of the intestine in disease initiation and progression [24]. Moreover, the epithelial cells in the small intestine appear to adapt to high fat diets also by increasing fatty acid oxidation through an upregulation of genes encoding for enzymes involved in β-and ωoxidation [21,25]. Increased lipoprotein secretion and increased fatty acid degradation may be taken as defense mechanisms to counteract the lipotoxic effect of high fat diets on intestinal cells [26], characterized by increased apoptosis rates, as found in rats receiving a high fat diet [27].

Since cholesterol and phospholipids are essential components of chylomicron assembly and since intestinal lipoprotein secretion is increased upon high fat feeding, more cholesterol and phospholipids are needed for the epithelial processing of fat, which may cause metabolic adaptations on mRNA levels of genes involved in these pathways. Although the effects of a high fat diet on cholesterol transporter gene expression in mice have already been described in a previous study [28], the diet used did not contain any cholesterol. Since a typical Western-style diet delivers fat mainly from animal sources, and thus also cholesterol, we aimed at assessing its effects on intestinal and hepatic cholesterol and phospholipid metabolism. For this, C57Bl/6 N mice were fed for 12 weeks either a cholesterol-free high carbohydrate control diet (C), comprising 4.2% fat (w/w), or a Western diet (W), with 34% fat (w/w) and 0.03% (w/w) cholesterol. We analyzed clinical chemistry parameters, assessed sterol balance and determined changes in gene expression profiles in intestine and liver. Despite a greatly elevated dietary cholesterol intake and cholesterolemia, small intestine and liver of mice fed the Western diet showed decreased levels of cholesterol, with changes in gene expression suggesting an increased cholesterol synthesis and an enhanced retrograde uptake. In addition, changes in the quantity and spectrum of different phosphatidylcholine (PC) species indicate that phospholipid metabolism is altered as well, most likely also to meet the increased demand for intestinal CM and hepatic VLDL secretion.

## Methods

### Ethics statement

All procedures applied throughout this study were conducted according to the German guidelines for animal care and approved by the Bavarian state ethics committee (Regierung von Oberbayern) according to §8 Abs.1 Tierschutzgesetz under the reference number 209.1/211-2531-41/03.

### Animals and sample collection

Conventionally raised eight-week-old male C57Bl/6NCrl mice (Charles River Laboratories) were housed individually in a light- and temperature-controlled facility (lights on 7 *a.m*. -7 *p.m*., 22°C) and had free access to water and food. They were fed a standard laboratory chow (Ssniff GmbH, cat. no. V1534) for two weeks and thereafter divided into two groups with similar mean body weights (n = 12). Mice were then fed group-specific pellet diets (control; Western) (Ssniff GmbH, cat. no. E15000-04 and E15741-34, respectively). The composition of the experimental diets is shown in Table 1. Throughout the feeding trial, body weight, food and water consumption were recorded once per week. Energy intake was corrected for spilled food, collected under metal grids placed below the food containers.

**Table 1:** Diet composition^*a*^

	Control	Western diet
**GE (MJ/kg)**	18.0	25.2

**ME (MJ/kg)**	15.2	21.4

**% protein**	23	19

**% fat**	11	60

**% carbohydrates**	66	21

**Casein**	240	276.9

**Corn starch mod**.	498	-

**Maltodextrin**	-	158

**Glucose**	100	-

**Sucrose**	-	80

**Cellulose**	50	60

**Vitamin premix**	10	12

**Mineral/trace elements**	60	61

**L-Cystine**	-	3.5

**Choline chloride**	2	2.5

**Salt (NaCl)**	-	1

**Butylhydroxytoluol**	-	0.1

**Beef tallow (premier jus)**	-	310

**Soybean oil**	40	30

**Cholesterol**	0	290

^*a *^Nutrient composition is expressed in g/kg except cholesterol which is provided as mg/kg. Abbreviations: *GE *gross energy; *ME *metabolizable energy calculated with the Atwater factors.

From days 4 to 11, 46 to 53 and 74 to 81, feces produced by five mice of each group were collected, dried at 50°C to constant weight and ground. Gross energy was determined using an isoperibol bomb calorimeter (model number 6300, Parr Instrument GmbH), with benzoic acid used as a standard.

After 12 weeks, mice in a non-fasting state were anesthetized using isoflurane and blood was collected from the retro-orbital sinus. Mice were then killed by cervical dislocation. Tissues were harvested at the same time of the light period (between 9 and 12 *a.m*.) for both groups to avoid diurnal variability. The small intestine was divided into two equal parts along the longitudinal axis (proximal and distal), mucosa was scraped off, snap-frozen in liquid nitrogen and stored at -80°C until further processing. Liver was collected, weighed and snap-frozen in liquid nitrogen.

### Glucose tolerance test

After 9 weeks of feeding, mice were subjected to a glucose tolerance test. After 14 hours of food deprivation, mice were injected with a 20% glucose solution (B. Braun Melsungen AG) intraperitoneally (10 ml/kg of body weight) and blood glucose was measured from the tail vein 0, 15, 30, 60 and 120 minutes after the injection using an Accu-Check blood glucose meter (Roche Diagnostics).

### Serum and tissue analysis

Serum cholesterol, glucose, HDL cholesterol and TG were determined using Piccolo^® ^Lipid Panel Plus Reagent Discs and a Piccolo Blood Chemistry Analyzer (Hitado Diagnostic Systems). Serum insulin was determined using an Ultra Sensitive Mouse Insulin ELISA kit (Crystal Chem Inc.), according to the manufacturer's instructions. Inter- and intra-assay CV were generally ≤ 10%.

For determination of hepatic and intestinal TG and PL concentration, tissues were ground in liquid nitrogen and dissolved in 0.9% NaCl. Samples were centrifuged for 10 min at 10 000 g and PL concentration was determined using a commercial enzymatic colorimetric kit, following the manufacturer's instructions (Phospholipids C, Wako Chemicals GmbH). TG were extracted from the samples as follows: after centrifugation (10 min, 10 000 g), supernatants were incubated in alcoholic KOH (30 min, 70°C), 0.15 mol/l magnesium sulfate was added to the solution and after centrifugation (10 min, 10 000 g), TG concentration was determined using a commercial enzymatic colorimetric kit, following the manufacturer's instructions (Triglycerides liquicolor^mono^, Human GmbH). Hepatic and intestinal cholesterol concentration was determined using a commercial enzymatic colorimetric kit, following the manufacturer's instructions (Cholesterol/Cholesteryl Ester Quantitation Kit, Biocat GmbH).

For determination of hepatic and intestinal acylcarnitine, phosphatidylcholine (PC) and sphingolipid concentration, tissues were ground in liquid nitrogen and analytes were extracted using 60 μl MeOH per 10 mg homogenized tissue. Samples were vortexed, centrifuged and the assay was performed in 10 μl of the supernatant using the *AbsoluteIDQ kit *(Biocrates Life Sciences AG), as previously described [29]. Briefly, acylcarnitines, PC and sphingolipids were detected with LC-MS/MS (3200QTrap-LC/MS/MS, Applied Biosystems) using Multi Reaction Monitoring pairs. Samples were delivered to the mass spectrometer by flow injection analysis method. The analytical process was performed using the MetIQ software package, an integral part of the *AbsoluteIDQ kit*.

### Fecal neutral sterol and bile acids determination

Sterol analysis in fecal samples was performed as previously described [30]. Coprostanol and cholesterol were summarized as total sterols. Epicoprostanol and coprostanone were below the limit of detection. Bile acids in fecal samples were determined as previously described [31] with minor modifications for murine feces samples. After extraction of bile acids, an internal standard was added (23nor-cholic acid, 30 μg) and after methylation, silylation and drying under a nitrogen stream, the residue was re-dissolved in 200 μl decane. The standard substances of deoxycholic acid (DCA) and cholic acid (CA) were purchased from Sigma but 12keto-DCA, 23nor-CA and alpha/omega-muricholic acid (alpha-MCA, omega-MCA) were purchased from Steraloids Inc.. The mass spectrometric detection was realized in multi ion current (23nor-CA: m/z = 253.20 amu; DCA: m/z = 255.30 amu; alpha-MCA: m/z = 403.00 amu, CA: m/z = 343.15 amu; 12keto-DCA: m/z = 231.25 amu; omega-MCA: m/z = 195.05 amu).

### RNA isolation

Total RNA from the upper and lower small intestine and from the liver was isolated using Trizol reagent (Invitrogen) until the ethanol precipitation step and further purified using the QIAGEN RNeasy Mini Kit spin columns (QIAGEN GmbH). RNA concentration and purity were measured on a NanoDrop ND-1000 UV-vis spectrophotometer (NanoDrop Technologies).

### Gene Chip expression array hybridization

Total RNA was reverse-transcribed and the corresponding cRNA was biotinylated and fragmented following the original protocol of Affymetrix (Affymetrix Inc.). For each experimental group, 6 biological replicates were hybridized overnight on The Gene Chip^® ^3' Expression Arrays (Affymetrix), customized for NuGO (The European Nutrigenomics Organization). A more detailed description of the platform can be found on the Gene Expression Omnibus, accession number GPL7441. The arrays were then washed and scanned following the instructions of the provider. A total of 24 arrays were hybridized. Detailed methods for the labeling and subsequent hybridizations to the arrays are provided in the eukaryotic section of the GeneChip Expression Analysis Technical Manual from Affymetrix.

### Transcriptome data analysis and statistics

The quality of the data was analyzed by a Bioconductor [32] and R based method in the Nutrigenomics Organisation NuGO Array Pipeline [33]. Expression levels of probe sets were normalized by GCRMA [34], using M-estimators for summarization. Differentially expressed probe sets were identified using Limma [35]. Custom CDF version 14 was used for annotation. Comparisons were made between the 2 groups and probe sets that showed a q-value ≤ 0.05 were considered significantly regulated. Array data have been submitted to the Gene Expression Omnibus under the accession number GSE29748.

Overrepresentation of gene ontology (GO) Biological Process subsets was made using an ErmineJ overrepresentation analysis [36]. Only genes with a p-value below 0.0025 and GO subsets containing between 8 and 125 genes were included in the analysis. GO subsets with a false discovery rate ≤ 0.05 were considered significantly regulated.

Heat map diagrams displaying standard scores of signal intensities of selected genes were made using the Genesis software [37] by applying hierarchical clustering. Only genes belonging to GO Biological Process subsets with a false discovery rate ≤ 0.05 following overrepresentation analysis with ErmineJ were included in the analysis.

### cDNA synthesis and real-time quantitative PCR

For each liver sample, 10 ng of isolated total RNA were used for real-time quantitative PCR (qPCR) using the QuantiTect^® ^SYBR Green RT-PCR kit (Qiagen GmbH) on a Mastercycler ep realplex apparatus (Eppendorf), following the suppliers' protocols. Gene sequences were retrieved from the database Ensembl http://www.ensembl.org/ and designed primers were tested for specificity using BLAST analysis http://blast.ncbi.nlm.nih.gov/Blast.cgi, melting curve analysis following qPCR and visualization on a 2% agarose gel. Primer sequences are shown in Additional file 1: Table S1. The following thermal cycling conditions were used: 1 cycle at 50°C for 30 min (cDNA synthesis), 1 cycle at 95°C for 15 min (RT enzyme inactivation), 40 cycles at 95°C for 15 s, 61°C for 30 s and 72°C for 30 s, followed by melting curve analysis (1.75°C/min). Cq-values were retrieved from the realplex 2.0 software (Eppendorf) and analyzed by the 2^-ΔΔCq ^method using the geometric mean of the housekeeping genes *glyceraldehyde-3-phosphate dehydrogenase *(*Gapdh*), *β-Actin *and *hypoxanthine guanine phosphoribosyl transferase *(*Hprt*) to normalize the data [38,39].

### Statistical analysis

For all groups, data were expressed as mean ± SEM. Statistical analyses were performed using the Prism 4 software (GraphPad Software). Prior to Student's t-test, data were tested for normal distribution and equality of variances. In the case of inhomogeneous variances, Welch's correction was applied to Student's t-test. Differences in weight gain, food and water intake, digested energy, fecal neutral sterol and bile acids output and daily sterol balance over the feeding period were tested by using the MIXED procedure in SAS (Version 9.2; SAS Institute Inc.) with time as a repeated factor [40]. The variables studied were subjected to 7 covariance structures: unstructured covariance, compound symmetry, autoregressive order one (AR(1)), autoregressive moving average order one (ARMA(1,1)), heterogeneous compound symmetry (CSH), heterogeneous autoregressive order one (ARH(1)) and Toeplitz. The goodness of fit of the models was compared using the Bayesian information criterion. Tukey's test was used as post-hoc test. Differences in hepatic and intestinal acylcarnitine, PC and sphingolipid levels were tested by Student's t-test with the Benjamini-Hochberg correction, using the R version 2.9.2 (R Foundation of Statistical Computing). For all tests, the bilateral alpha risk was α = 0.05.

## Results

### Western diet feeding led to obesity, hyperglycemia, hyperinsulinemia and elevated blood cholesterol levels

After 12 weeks on a Western diet, mice presented the expected hallmarks of obesity. Data on final body weight, as well as cumulative food, energy, water and macronutrient intake is provided in Additional file 2: Table S2. Digested energy was calculated as [energy intake] - [energy remaining in feces]. Body weight development is given in Additional file 3: Figure S1. A glucose tolerance test carried out at week 9 of the feeding trial revealed a delayed blood glucose clearance in the obese mice as compared to the control mice (Additional file 4: Figure S2A and S2B). Blood collected in the non-fasting state right before sacrifice revealed a hyperglycemia, a 6-fold increase in mean serum insulin concentration, a 2-fold increase in mean serum cholesterol concentration and a 70% increase in plasma HDL-cholesterol levels in the mice on the Western diet (Additional file 5: Table S3).

### Mice on Western diet displayed increased fecal neutral sterol content

**Figure 1:**
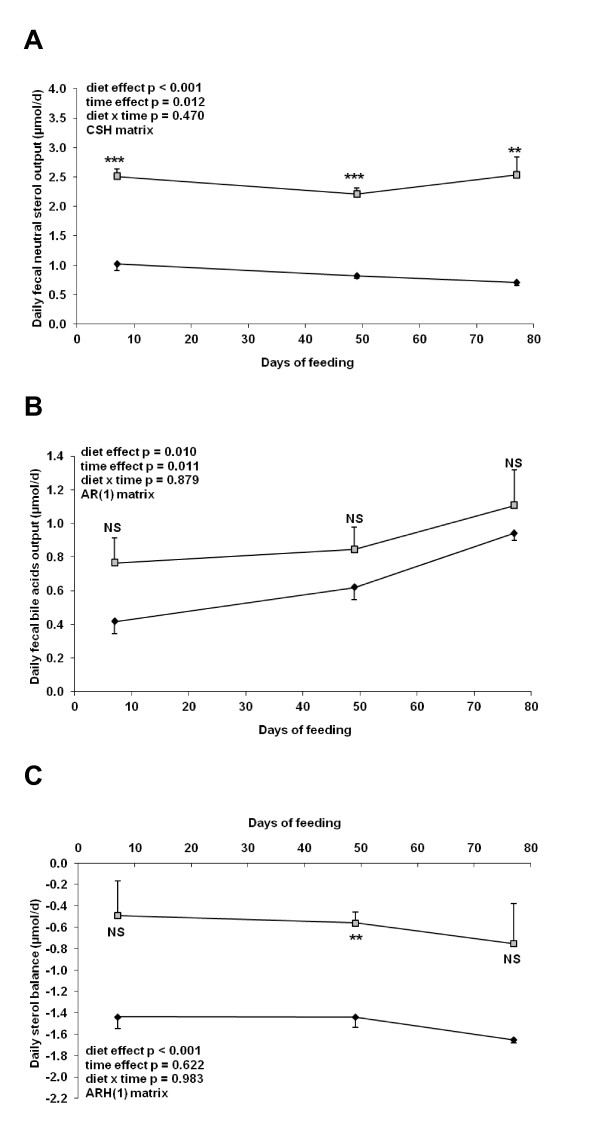
**Sterol balance data obtained from mice fed the different diets for 12 weeks**. Feces were collected at three time points during the feeding trial and neutral sterol and bile acids content was measured by gas chromatography-mass spectrometry **A**: Daily fecal neutral sterol output. **B**: Daily fecal bile acids output. **C**: Daily sterol balance measured by subtracting fecal neutral sterol and bile acids output from cholesterol intake. Symbols: black diamonds, control diet; grey squares, Western diet. Data are presented as mean ± SEM (n = 5). ** *p *< 0.01; *** *p *< 0.001, NS: not significant.

Feces from five mice per group were collected from days 4 to 11, 46 to 53 and 74 to 81 and analyzed for neutral sterol and bile acids content. Mice receiving the Western diet exhibited an increase in fecal neutral sterol output (Figure 1A). Fecal bile acid losses were also increased in the mice fed the Western diet (Figure 1B) but according to Tukey's test, this did not reach significance. We also calculated a daily sterol balance in each group by subtracting the amount of neutral sterol and bile acids lost in the feces to the dietary cholesterol intake.

This balance did not include beta muricholic acid and steroid hormones derivatives. Interestingly, mice fed the Western diet displayed a negative sterol balance, losing between 0.50 to 0.75 μmol of cholesterol per day. Mice fed the control diet showed an even more pronounced negative sterol balance, excreting between 1.45 to 1.65 μmol of cholesterol per day, although this was only significantly different from mice fed the Western diet between days 46 and 53 (Figure 1C).

### Obese mice displayed decreased intestinal and hepatic cholesterol levels

**Figure 2:**
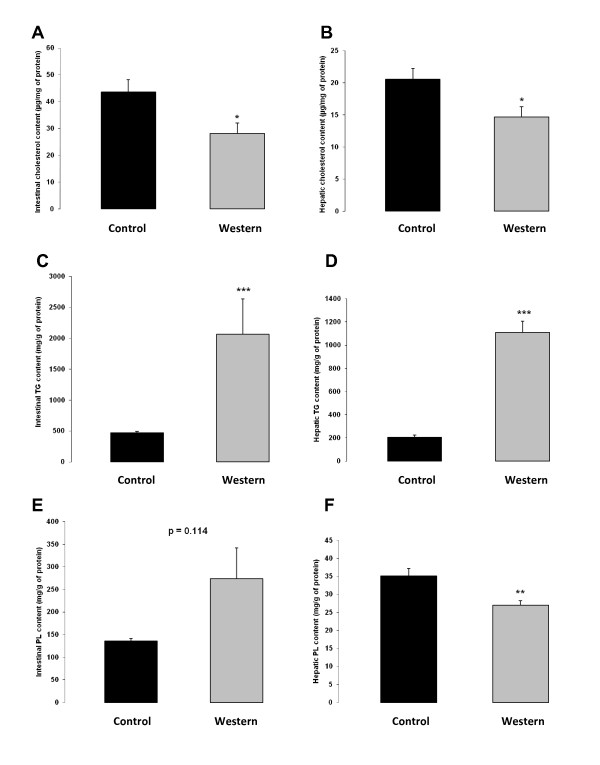
**Cholesterol, TG and PL content in intestine and liver of mice fed the different diets for 12 weeks**. **A**: Cholesterol concentration in the upper small intestine (n = 5). **B**: Cholesterol concentration in the liver (n = 12). **C**: TG concentration in the upper small intestine (n = 5-6). **D**: TG concentration in the liver (n = 11-12). E: PL concentration in the upper small intestine (n = 5-6). F: PL concentration in the liver (n = 11-12). Control diet: black bar; Western diet: grey bar. Data are presented as mean ± SEM. * *p *< 0.05; ** *p *< 0.01; *** *p *< 0.001.

Despite a much greater dietary cholesterol intake (Additional file 2: Table S2), mice fed the Western diet displayed a 35% reduction (*p *= 0.035) in intestinal cholesterol concentration and a 29% reduction (*p *= 0.019) in hepatic cholesterol concentration (Figure 2A and 2B). In addition, the obese mice presented a massive accumulation of intra-intestinal and intrahepatic TG with a 4.4- and a 5.3-fold increase respectively, as compared to control mice (Figure 2C and 2D). We also observed a marginally increased PL concentration in intestinal samples from obese mice (*p *= 0.114) (Figure 2E), whereas in liver samples a 23% decrease (*p *= 0.004) in PL content was detected (Figure 2F).

### Cholesterol transporter genes showed reduced expression levels while cholesterol synthesis genes showed increased expression levels in the small intestine of obese mice

**Figure 3:**
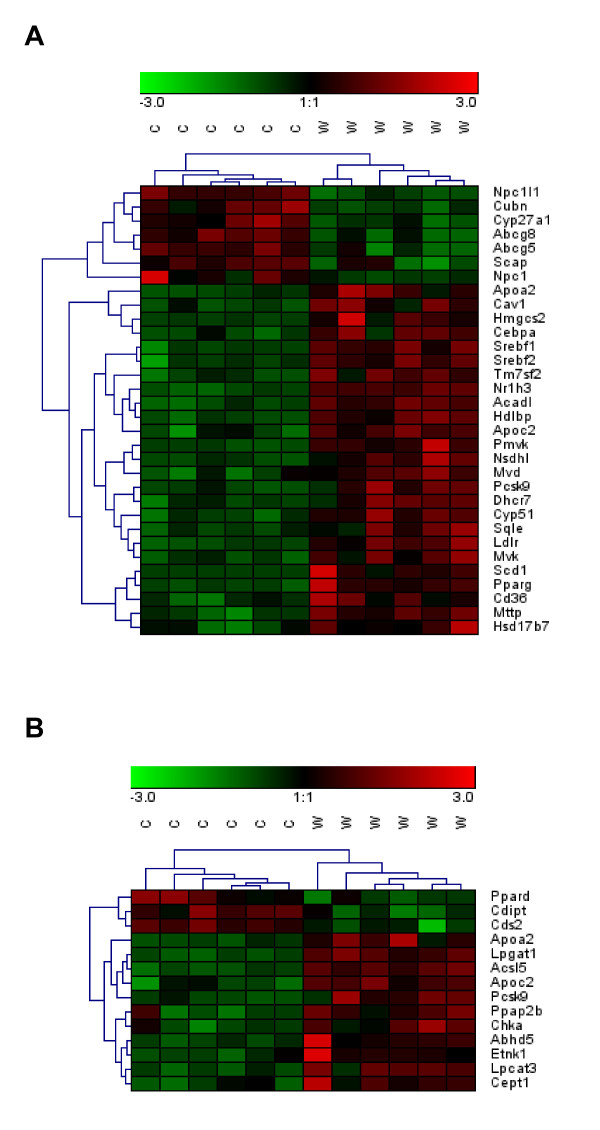
**Heat map diagrams of differentially expressed genes in the small intestine upon Western diet feeding**. A: Standard scores of differentially expressed genes related to cholesterol metabolism (GO Biological Processes: cholesterol metabolic process, cholesterol biosynthetic process, cholesterol transport, cholesterol homeostasis, positive regulation of cholesterol efflux, regulation of cholesterol efflux, cholesterol efflux, regulation of cholesterol metabolic process, regulation of cholesterol storage, regulation of cholesterol biosynthetic process, reverse cholesterol transport). B: Standard scores of differentially expressed genes related to PL metabolism (GO Biological Processes: PL metabolic process, PL biosynthetic process, PL catabolic process, PL efflux, PL transport). Capital letters indicate: C, control; W, Western diet. Differentially expressed genes with a q-value ≤ 0.05 were included in the analysis. Green and red indicate down- and up-regulation of gene expression, respectively.

Expression levels of genes encoding proteins directly involved in cholesterol transport or metabolism in the small intestine, obtained from microarray analysis, are shown in Table 2 and visualized in Figure 3A. These genes were much more affected by dietary treatment in the upper than in the lower part of the small intestine and therefore, only the changes observed in the duodenum and the proximal jejunum are presented. A complete list of all genes analyzed and their associated fold changes and q-values in the upper and lower small intestine are given in Additional file 6: Table S4 and Additional file 7: Table S5 respectively. Overrepresentation analysis of GO Biological Processes revealed as well several gene subsets involved in cholesterol transport or metabolism (Additional file 8: Table S6). The cholesterol efflux transporters *Abcg5 *and -*8 *and the cholesterol absorption transporter *Npc1l1 *showed reduced mRNA levels in mice on Western diet as compared to control mice. *Abca1*, a cholesterol efflux transporter located at the basolateral side of the enterocyte, was not affected by the dietary treatment. In mice fed the Western diet, several genes relevant for the biosynthesis of cholesterol (*Pmvk, Mvk, Mvd, Sqle, Cyp51, Nsdhl, Tm7sf2, Dhcr7, Hsd17b7*) were found consistently upregulated in the intestinal tissue. However, we did not observe any regulation for *Hmgcr*, the gene encoding the rate-limiting enzyme in the cholesterol biosynthesis pathway. *Srebp-2*, a nuclear factor regulating the expression of genes involved in cholesterol synthesis, was significantly upregulated in the intestine of obese mice. We also observed increased mRNA levels for *Apoa2, Apoc2 *and the *microsomal triglyceride transfer protein, Mttp*, all involved in chylomicron assembly. A strong downregulation of *Cyp27a1*, which could translate into a reduced conversion of cholesterol to 27-hydroxycholesterol was also observed. Nonetheless, *LXRα*, a nuclear factor activated by 27-hydroxycholesterol, was also upregulated as well as the *LDL-receptor*. Moreover, mRNA levels of several genes encoding proteins involved in fatty acid β-and ω-oxidation were increased. The most impressive regulation was found for *Scd1*, the *stearoyl-coenzyme A desaturase 1*, a lipogenic enzyme catalyzing the formation of monounsaturated fatty acids (MUFA), which serve as components of membrane PL, TG and cholesterol esters. In addition, *Ces1d *and *Ces1g*, two genes encoding for carboxylesterases, displayed a strong downregulation.

**Table 2:** Effect of a chronic Western diet on the expression of genes related to cholesterol and lipid metabolism in the small intestine ^*a*^

Symbol	Gene name	FC	q-value
**Abca1**	ATP-binding cassette, sub-family A, 1	-1.13	0.466

**Abcg5**	ATP-binding cassette, sub-family G, 5	-1.48	0.006

**Abcg8**	ATP-binding cassette, sub-family G, 8	-2.35	0.002

**Acaa2**	acetyl-Coenzyme A acyltransferase 2	1.67	0.010

**Apoa2**	Apolipoprotein A-II	3.15	0.001

**Apoc2**	Apolipoprotein C-II	1.36	0.006

**Cav1**	Caveolin 1	1.6	0.006

**CD36**	CD36 antigen	2.11	0.020

**Ces1d**	Carboxylesterase 1D	-6.13	< 0.001

**Ces1g**	Carboxylesterase 1G	-5.46	< 0.001

**Cpt1a**	Carnitine palmitoyltransferase 1a, liver	1.55	0.044

**Cyp27a1**	Cytochrome P450, family 27, subfamily a, polypeptide 1	-2.57	0.004

**Cyp51**	Cytochrome P450, family 51	2.03	0.001

**Dhcr7**	7-dehydrocholesterol reductase	1.48	0.011

**Hmgcl**	3-hydroxy-3-methylglutaryl-Coenzyme A lyase	1.57	0.024

**Hmgcr**	3-hydroxy-3-methylglutaryl-Coenzyme A reductase	1.02	0.748

**Hmgcs2**	3-hydroxy-3-methylglutaryl-Coenzyme A synthase 2	8.07	< 0.001

**Hsd17b7**	Hydroxysteroid (17-beta) dehydrogenase 7	1.32	0.045

**Idh1**	Isocitrate dehydrogenase 1 (NADP+), soluble	1.31	0.010

**LDLr**	LDL receptor	4.29	< 0.001

**LXRα**	Liver × receptor alpha	1.77	0.003

**LXRβ**	Liver × receptor beta	-1.06	0.646

**Me1**	Malic enzyme 1, NADP(+)-dependent, cytosolic	3.13	< 0.001

**Mttp**	Microsomal triglyceride transfer protein	1.18	0.016

**Mvd**	Mevalonate decarboxylase	1.33	0.012

**Mvk**	Mevalonate kinase	1.22	0.017

**Npc1l1**	Niemann-Pick C1-like protein 1	-2.02	< 0.001

**Nsdhl**	NAD(P) dependent steroid dehydrogenase-like	1.45	0.004

**Pcsk9**	Proprotein convertase subtilisin/kexin type 9	1.65	0.007

**Pmvk**	Phosphomevalonate kinase	2.46	< 0.001

**Scarb1**	Scavenger receptor class B, member 1	1.4	0.447

**Scd1**	Stearoyl-Coenzyme A desaturase 1	90.7	< 0.001

**Scd2**	Stearoyl-Coenzyme A desaturase 2	6.50	< 0.001

**Slc25a1**	Solute carrier family 25 (mitochondrial carrier, citrate transporter), member 1	2.04	< 0.001

**Slc27a4**	solute carrier family 27 (fatty acid transporter), member 4	-1.12	0.209

**Sqle**	Squalene epoxidase	2.11	0.011

**Srebp-2**	Sterol regulatory element binding factor 2	1.59	0.004

**Tm7sf2**	Transmembrane 7 superfamily member 2	1.52	0.003

^*a *^Abbreviations: *FC *fold change (Western diet vs. control).

To assess whether these changes were restricted to intestinal tissue or similarly occur in the liver, we used qPCR to determine transcript levels of the preselected target genes involved in cholesterol metabolism (Table 3). In the liver, the gene encoding for Hmgcr as well as Srebp-2, Cyp51 and Dhcr7 were significantly upregulated in mice on the Western diet when compared to the control group.

**Table 3:** Effect of a chronic Western diet on the expression of genes related to cholesterol metabolism in the liver^*a*^

Symbol	Gene name	FC	p-value
**Cyp51**	Cytochrome P450, family 51	1.80 ± 0.28	0.056

**Dhcr7**	7-dehydrocholesterol reductase	1.72 ± 0.23	0.025

**Hmgcr**	3-hydroxy-3-methylglutaryl-Coenzyme A reductase	2.23 ± 0.48	0.033

**Pmvk**	Phosphomevalonate kinase	1.22 ± 0.16	0.378

**Srebp-2**	Sterol regulatory element binding factor 2	1.37 ± 0.08	0.009

*^a ^*Abbreviations: *FC *fold change (Western diet vs. control)

### Liver and small intestine exhibited changes in phospholipid status and metabolism

Significant changes in expression levels of genes encoding proteins directly involved in PL processing in the small intestine are shown in Table 4 and visualized in Figure 3B. Whereas *CDP-diacylglycerol synthase 2 *and *CDP-diacylglycerol-inositol 3-phosphatidyltransferase*, as well as *lysophosphatidylcholine acyltransferase 1 *and *3 *showed only modestly altered mRNA levels, *lysophosphatidylglycerol acyltransferase 1 *mRNA level was increased 1.52 fold and, most prominently, *phosphatidic acid phosphatase type 2B *exhibited a 2.51-fold upregulation, while *scramblase 2 *showed a 7.8-fold and *scramblase 4 *even a 24-fold increased mRNA level.

**Table 4:** Effect of a chronic Western diet on the expression of genes related to phospholipid metabolism (based on GO classification) in the small intestine^*a*^

Symbol	Gene name	FC	q-value
**Cdipt**	CDP-diacylglycerol--inositol 3-phosphatidyltransferase (phosphatidylinositol synthase)	-1.29	0.015

**Cds2**	CDP-diacylglycerol synthase (phosphatidate cytidylyltransferase) 2	-1.29	0.020

**Cept1**	Choline/ethanolaminephosphotransferase 1	1.26	0.030

**Chka**	Choline kinase alpha	1.90	0.027

**Lpcat3**	Lysophosphatidylcholine acyltransferase 3	1.39	0.005

**Lpgat1**	Lysophosphatidylglycerol acyltransferase 1	1.52	< 0.001

**Plscr2**	Phospholipid scramblase 2	7.83	< 0.001

**Plscr4**	Phospholipid scramblase 4	24.31	< 0.001

**Ppap2b**	Phosphatidic acid phosphatase type 2B	2.51	0.022

^*a *^Abbreviations: *FC *fold change (Western diet vs. control)

**Figure 4:**
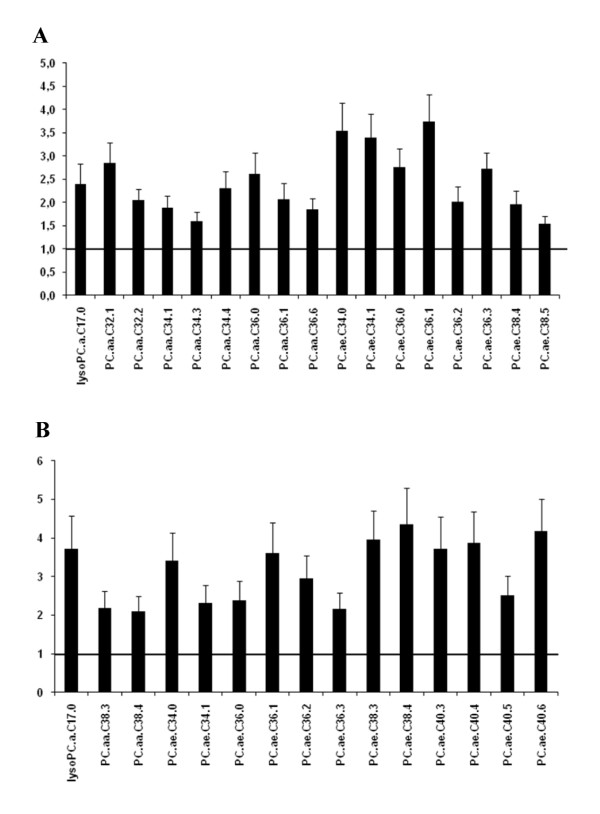
**Effect of a chronic Western diet on the level of phosphatidylcholine species**. Hepatic and intestinal PC levels were detected using LC-MS/MS. A: Significantly regulated PC in the small intestine (n = 6). B: Significantly regulated PC in the liver (n = 6). Data are presented as fold change (Western diet versus control) ± SEM. Abbreviations: PC.aa.: phosphatidylcholine diacyl; PC.ae.: phosphatidylcholine acyl-alkyl; lysoPC.a.: lysophosphatidylcholine acyl.

Based on LC-MS/MS analysis, a variety of changes in intestinal and hepatic phospholipids were identified. The fold changes of significantly regulated phosphatidylcholine (PC) species in tissue samples of mice fed the Western diet compared to mice fed the control diet are displayed in Figure 4. A complete list of all metabolites analyzed, including acylcarnitines and sphingolipids, and their respective concentrations in the small intestine and liver samples is given in Additional file 9: Table S7. Among the 84 PC species analyzed, 17 showed significantly increased concentrations in the small intestine and 15 (up to four-fold) in the liver of mice fed the Western diet compared to the control group.

## Discussion

The main finding of this study is that obese mice fed a Western-style high fat diet containing cholesterol displayed reduced cholesterol levels in intestine and liver, despite a plasma hypercholesterolemia, when compared to mice given a cholesterol-free high carbohydrate diet. Not only did the mice on the Western diet exhibit phenotypic changes towards a metabolic syndrome, such as impaired glucose clearance, but also major adaptive changes in cholesterol and phospholipid metabolism.

**Figure 5:**
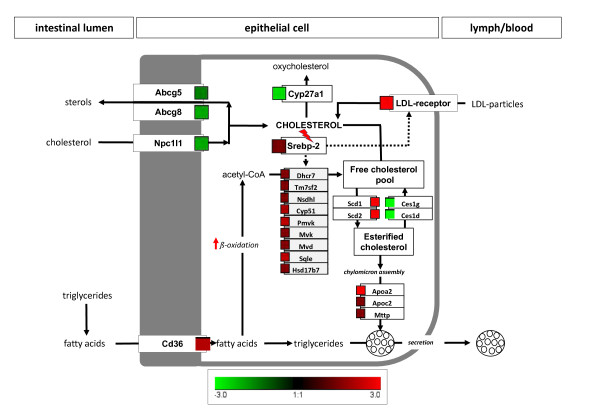
**Effect of a chronic Western diet on the expression of genes related to cholesterol metabolism in the small intestine**. Fold changes are displayed next to differentially expressed genes with color code provided. Red squares indicate upregulated genes; green squares indicate downregulated genes. Cholesterol concentration was found to be decreased whereas TG concentration was found to be increased in mice receiving the Western diet. Abbreviations and fold changes are listed in Table 2.

Proper fat digestion and absorption in the small intestine requires luminal bile acids and PL for formation of micelles. Incorporation of TG into CM after reassembly in the enterocytes also requires large quantities of PL and cholesterol. Chronic high fat feeding consequently increases the needs of the small intestine for additional cholesterol, PL and bile acids for processing and secretion of the fat into circulation. Although Western diets based on animal lipid sources provide extra cholesterol, this did not seem to be sufficient to meet the increased demands of the intestine. Based on the microarray data, we provide evidence that the subsequent fall in tissue cholesterol levels may initiate changes in gene expression that can be interpreted as an increase in *de novo *cholesterol synthesis, a decreased cholesterol efflux into the intestinal lumen and an increased cholesterol uptake from circulation into the epithelium via LDL and the LDL-receptor. These changes are summarized schematically in Figure 5.

Evidence for an increased *de novo *synthesis of cholesterol in the intestine is derived from increased mRNA levels of the *mevalonate kinase *(*Mvk*), the *phosphomevalonate kinase *(*Pmvk*), the *mevalonate decarboxylase *(*Mvd*), the *squalene epoxidase *(*Sqle*), the *cytochrome P450, family 51 *(*Cyp51*), the *7-dehydrocholesterol reductase *(*Dhcr7*), the *hydroxysteroid (17-beta) dehydrogenase 7 *(*Hsd17b7*), the *NAD(P) dependent steroid dehydrogenase-like *(*Nsdhl*) and the *transmembrane 7 superfamily member 2 *(*Tm7sf2*) genes. *Nsdhl *encodes a sterol dehydrogenase while *Tm7sf2 *encodes a sterol reductase, both involved in post-squalene cholesterol biosynthesis [41-43]. Although *3-hydroxy-3-methylglutaryl-coenzyme A reductase *(*Hmgcr*), the rate-controlling enzyme in cholesterol synthesis, did not exhibit any significant changes in mRNA levels upon Western diet feeding, it is known to also be extensively regulated at the post-transcriptional level [28]. The precursor for cholesterol synthesis is acetyl-CoA, either provided from pyruvate via glycolysis, or derived from fatty acid oxidation in mitochondria and shuttled into the cytosol as citrate with the concomitant release of acetyl-CoA via the ATP-citrate lyase. Amongst the genes needed for fatty acid import into mitochondria and ß-oxidation, increased mRNA levels of the *carnitinepalmitoyltransferase *(*Cpt1a*), and the *3-hydroxy-3-methylglutaryl-coenzyme A synthase 2 *(*Hmgcs2*), with an 8-fold increase in mRNA levels, were identified. In addition, *3-hydroxy-3methylglutaryl-coenzyme A lyase *(*Hmgcl*) and *acetyl-coenzyme A acyltransferase 2 *(*Acaa2*) were found to be upregulated, indicative also for an increase in fatty acid oxidation. Increased mRNA levels of the *isocitrate dehydrogenase 1 *(*Idh1*) and the citrate exporter in the inner mitochondrial membrane (*Slc25a1*) may indicate a simultaneous increase in citric acid cycle activity and enhanced delivery of acetyl-CoA for cytosolic cholesterol synthesis. The increased demand of NADPH for the reductive cholesterol biosynthesis may be met by an increase in the expression of *cytosolic malic enzyme *(*Me1*) that showed a 3-fold elevation in mRNA levels. Very similar findings, with corresponding changes in catalytic activities of malic enzyme, carnitine-palmitoyltransferase and ß-oxidation in obesity-prone C57Bl/6 J mice, were reported by Kondo et al. (24). Moreover, *Mttp, Apoa2 *and *Apoc2*, three genes involved in CM assembly, displayed elevated mRNA levels, indicative of an increase in CM formation upon high fat feeding. We also observed a 2-fold upregulation of *Cd36 *in mice fed the Western diet. Interestingly, it has recently been suggested that CD36 might act as a lipid sensor optimizing the formation of large CM in the small intestine [44].

Genes involved in the cholesterol biosynthesis pathway are primarily under the control of the membrane-bound transcription factor sterol regulatory element-binding protein 2 (Srebp-2) [45]. When the demand for intracellular cholesterol rises, the Srebp-2 pathway is activated and causes increased transcription of specific target genes [46]. We observed elevated levels of *Srebp-2 *in the intestine of the mice fed the Western diet, suggesting an adaptive increase in cholesterol synthesis. The mRNA levels of the *LDL-receptor*, another Srebp-2 target gene, were 4-fold higher in mice on the Western diet. This suggests an increased re-uptake from circulating LDL to meet the elevated cholesterol demand of the tissue [47]. The downregulation of *Abcg5 *and *Abcg8*, both in the upper and lower small intestine, which act as cholesterol efflux transporters in the apical membrane of enterocytes, may as well be interpreted as such a compensatory mechanism to prevent cholesterol losses. These transporters have recently been associated with trans-intestinal cholesterol excretion (TICE) which appears to significantly contribute to fecal neutral sterol loss in mice [48]. Although dietary modifications were shown to alter cholesterol secretion in the intestine, a high cholesterol diet failed to affect TICE [49]. Our data on the downregulation of *Abcg5/8 *confirm similar findings in a diet-induced obesity mouse model with a high fat but cholesterol-free diet [21]. This may suggest that high fat or Western-style diets do regulate these cholesterol efflux transporters more or less independently of dietary cholesterol. This could translate into a reduced TICE which would contribute to the observed decrease in net cholesterol excretion (Figure 1C) and the elevated circulating cholesterol level (Additional file 5: Table S3) in the mice fed the Western diet. Surprisingly, the gene encoding for Npc1l1 exhibited a 2-fold downregulation in the present study. This protein is believed to be the prime import transporter mediating cholesterol absorption. However, it has been shown in mice that a cholesterol-rich diet reduces *Npc1l1 *expression [11] and a cholesterol-free high fat diet similarly decreases *Npc1l1 *expression as well as cholesterol absorption when compared to mice fed a cholesterol-free but low fat diet [28]. Amongst the enzymes that mediate cholesterol degradation, Cyp27a1 plays a prominent role and its gene was found to be downregulated, around 3-fold, in the Western diet fed mice, suggesting an additional inhibition of cholesterol breakdown. The most prominent regulation in gene expression was found for the *stearoyl-coenzyme A desaturase 1 *(*Scd1*), encoding for the rate-limiting enzyme in MUFA synthesis, whose expression levels were 90 times higher in the Western diet fed mice. MUFA are a key component to the formation of TG, cholesterol esters and PL [50] and it has been shown that the synthesis of cholesterol ester is actually dependent on MUFA produced by Scd1 [51]. Since esterified cholesterol is required for CM assembly, the huge increase in *Scd1 *expression may reflect the increased need of cholesteryl ester for CM secretion. *Scd2*, which encodes the same functional protein, was found to be strongly upregulated as well. Interestingly, *Ces1d *and *Ces1g*, two genes encoding carboxylesterases, were found to be strongly downregulated, -6.13 and -5.46 times respectively, suggesting an inhibition of the hydrolysis of cholesteryl ester to cholesterol [52].

Taken together, the changes in mRNA levels observed in the mice receiving a cholesterol-containing Western diet may be interpreted as an adaptive response to meet an increased cholesterol demand of the intestine for proper handling of large quantities of fat and for CM formation. The changes suggest a reduced luminal efflux of cholesterol, an increased LDL-receptor mediated reverse uptake, a reduced breakdown and an enhanced *de novo *synthesis. These findings confirm data reported by de Vogel-van den Bosch et al. [28] on changes in gene expression of intestinal cholesterol metabolism in mice fed a cholesterol-free high fat diet for 8 weeks. We here extent this observation by showing that even in the presence of cholesterol in the Western diet, tissue demands cannot be met and supply may simultaneously be increased via uptake of LDL particles and *de novo *synthesis, with increased fatty acid ß-oxidation providing the building blocks. Most of these effects are likely to be mediated through *Srebp-2*. We also observed similar changes in mRNA levels of selected genes such as *Srebp-2, Hmgcr, Cyp51 *and *Dhcr7 *in liver samples of the mice fed the Western diet, suggesting that hepatic cholesterol synthesis is increased as well. Under conditions of a high dietary fat intake, the liver also has an increased cholesterol demand for VLDL secretion and bile production and secretion. The observed steatohepatosis, a hallmark in rodent models of obesity, may demonstrate the restricted capacity for lipid export from the liver via VLDL. It should be noted that the Western diet provided around 3 energy% of fructose which may as well have contributed to the development of the steatohepatosis observed [53].

Lipid and cholesterol handling, both in the intestinal lumen and for transport via CM and other lipoproteins, requires PL. Numerous genes involved in PL homeostasis exhibited altered expression levels in the small intestine of the obese mice. For example, genes encoding phospholipid scramblases 2 and 4 were upregulated 7.8- and 24.3-fold respectively in mice fed the Western diet. Phospholipid scramblases represent a group of homologous ATP-independent bidirectional lipid translocators, involved in generation and maintenance of lipid asymmetry in the plasma membrane and are conserved in all eukaryotes [54,55]. Fat processing in intestinal cells causes, at least transiently, a rearrangement of plasma cell membranes, increases membrane synthesis and vesicular trafficking, with a need for a remodeling of all cellular membrane compartments [56,57]. Although the biological functions of the phospholipid scramblases 2 and 4 need to be determined, these changes may be taken as a signature of major alterations in PL metabolism in the intestinal epithelium, induced by Western diet feeding.

Analysis of total PL content in liver revealed significantly reduced levels in mice fed the Western diet, whereas changes in the small intestine did not reach significance. However, as demonstrated by Hicks et al., different tissues present unique PL signatures [58]. Hence, we analyzed the PL spectrum in intestinal and liver samples via LC-MS/MS. Phosphatidylcholine (PC) and phosphatidylethanolamine (PE) are the two prominent phospholipid classes [59] and amongst all PC species analyzed, 17 displayed markedly increased levels in the small intestine and 15 as well in the liver, although those mostly did not belong to the species with the highest concentrations. Despite unchanged (intestine) or reduced (liver) total PL content, major alterations in the spectrum of glycerophospholipids could be detected, particularly in PC species with carbon chain length of C32 and C34.

Furthermore, liver also exhibited alterations in PC species with longer carbon chains (C38 and C40). In essentially all mammalian cells, PC is synthesized almost exclusively through the CDP-choline pathway [59]. However, hepatocytes uniquely express a PE methyltransferase, which methylates PE to PC via three sequential steps [60]. Interestingly, DeLong et al. showed that PC derived from the PE methylation pathway were comprised of significantly more long chain, polyunsaturated fatty acids [61]. Thus, the higher levels of PC species with longer carbon chains found in the liver could originate most likely from an increased activity of the PE methylation pathway in hepatocytes. We also observed increased concentrations of PC diacyl 34:1 (PC.aa.34:1) in small intestine and increased concentrations of the analogue etherphospholipid PC acyl-alkyl 34:1 (PC.ae.34:1) in both small intestine and liver of mice fed the Western diet. Recently, Chakravarthy et al. demonstrated that PC.aa (16:0/18:1) is a natural ligand of the nuclear receptor peroxisome proliferator-activated receptor alpha (PPAR-α), which promotes fatty acid oxidation, lipid transport and ketogenesis in liver and intestine [62]. The increased levels of PC.aa.34:1 suggest that a PPAR-α dependent activation by this agonist could contribute as well to some of the changes observed in the small intestine of the mice on Western diet [63]. The role of the etherphospholipid PC.ae.34:1 in PPAR-α activation remains yet to be determined. Gene expression analysis in intestinal samples also revealed evidence for significant changes in PL metabolism. Whereas *choline kinase alpha *(*Chka*) as well as *choline/ethanolaminephosphotransferase 1 *(*Cept1*), 2 genes involved in the CDP-choline pathway, showed increased mRNA levels, *CDP-diacylglycerol synthase *and *phosphatidylinositol synthase *displayed reduced levels. *Lysophosphatidylcholine acyltransferase 3 *(*Lpcat3*), a gene involved in the conversion of lysoPC to PC and essential for membrane asymmetry and diversity [64], was also found to be upregulated in the Western diet group. Yet, lysophosphatidylcholine acyl C17:0 (lysoPC.a.17.0) concentrations were markedly increased in intestine and liver, suggesting an imbalance of phospholipase- and Lpcat3-mediated cleavage and re-esterification. The *phosphatidic acid phosphatase type 2B *showed a 2.51-fold increased mRNA level, which could lead to increased concentrations of diacylglycerol, which, together with the increase in *Cept1 *mRNA levels, suggests an increased synthesis of PC and PE, while the synthesis of phosphatidylinositol may be reduced. Although phospholipases were not found to change in expression levels, increased mRNA levels of *lysophosphatidylcholine acyltransferases 1 *and *3 *suggest an increased overall turnover of the different PC species in the tissues.

## Conclusions

In summary, in addition to obesity, impaired glucose tolerance and hepatic steatosis, a Western-style high fat diet, which usually contains larger quantities of cholesterol, also causes changes in enterohepatic cholesterol and PL status in C57Bl/6 N mice. Despite a higher dietary intake of cholesterol and increased serum cholesterol levels, tissue cholesterol levels in small intestine and liver were reduced when compared to lean mice fed a cholesterol-free high carbohydrate diet. Changes in gene expression suggest that the increased cholesterol demand of tissues for fat absorption and transport may be met by a) an increased export of acetyl-CoA from mitochondria and increased cytosolic cholesterol synthesis, b) a reduced breakdown of cholesterol, c) an increased reverse uptake of cholesterol via LDL and d) a reduced back-flux of cholesterol into the intestinal lumen. In addition, we demonstrate that intestine and liver show major changes in gene expression and in the levels of selected PC species, indicative for a) an increased PC synthesis via the CDPcholine pathway, b) an increased PE methylation pathway activity in the liver and c) alterations in membrane PL remodeling. The observed cholesterol paradox and alterations in PL metabolism call for more studies to identify the underlying molecular mechanisms by which Western-style high fat diets cause these changes.

## Abbreviations

ABCG5: ATP-binding cassette sub-family G member 5; ABCG8: ATP-binding cassette subfamily G member 8; AR(1): autoregressive order one; ARH(1): heterogeneous autoregressive order one; ARMA(1,1): autoregressive moving average order one; CA: cholic acid; CM: chylomicron; CSH: heterogeneous compound symmetry; DCA: deoxycholic acid; GO: gene ontology; Lpact3: Lysophosphatidylcholine acyltransferase 3; MCA: muricholic acid; MUFA: mono unsaturated fatty acids; NPC1L1: Niemann-Pick C1 Like Protein 1; PC: phosphatidylcholine; PC.aa.34:1: phosphatidylcholine diacyl 34:1; PC.ae.34:1: phosphatidylcholine acyl-alkyl 34:1; PE: phosphatidylethanolamine; PL: phospholipid; PPAR-α: peroxisome proliferator-activated receptor alpha; qPCR: real-time quantitative Polymerase Chain Reaction; TG: triacylglyceride; TICE: trans-intestinal cholesterol excretion; W: Western diet.

## Competing interests

The authors declare that they have no competing interests.

## Authors' contributions

CDe carried out the animal experiment, the transcriptomics study, analyzed data and drafted the manuscript; CDa carried out the qPCR; MS carried out the metabolomics study; SK and GJ carried out the fecal neutral sterol and bile acids determination; HD designed the study and drafted the manuscript. All authors read and approved the final manuscript.

## Supplementary Material

Additional file 1 **Table S1. Primer sequences**. Click here for file

Additional file 2 **Table S2. Effect of a chronic Western diet on final body weight, cumulative food, energy, water, macronutrient and cholesterol intake and energy assimilation**. Click here for file

Additional file 3 **Figure S1. Body weight development in mice fed the different diets**. Symbols: black diamonds, control diet; grey squares, Western diet. Data are presented as mean ± SEM (n = 12). ** *p *< 0.01; *** *p *< 0.001. Click here for file

Additional file 4 **Figure S2. Glucose tolerance in mice fed the different diets for 9 weeks. A**: A glucose tolerance test was carried out after 9 weeks of dietary intervention following 14 h of food deprivation. Blood was collected from the tail vein 0, 15, 30, 60 and 120 min after an intraperitoneal 20% glucose solution injection (10 ml/kg of body weight) and blood glucose was measured. Symbols: black diamonds, control diet; grey squares, Western diet. **B**: Area Under the Curve (AUC) calculated from the glucose tolerance test. Black bar: control diet; grey bar: Western diet. Data are presented as mean ± SEM (n = 12). * *p *< 0.05. Click here for file

Additional file 5 **Table S3. Effect of a chronic Western diet on selected blood parameters**. Click here for file

Additional file 6 **Table S4. Effect of a chronic Western diet on gene expression in the upper small intestine**. Data are presented by ascending q-values. Abbreviations: FC: fold change. Click here for file

Additional file 7 **Table S5. Effect of a chronic Western diet on gene expression in the lower small intestine**. Data are presented by ascending q-values. Abbreviations: FC: fold change. Click here for file

Additional file 8 **Table S6. Effect of a chronic Western diet on GO Biological Process subsets in the upper small intestine**. Data are presented by ascending corrected p-value. Click here for file

Additional file 9 **Table S7. Effect of a chronic Western diet on the level of selected phospholipids (PL)**. Hepatic and intestinal PC levels were detected using LC-MS/MS. Data are presented are mean in μmol/g of protein ± SEM. Abbreviations: C0: DL-carnitine; C2: acetyl-L-carnitine; C3: propionyl-L-carnitine; C4: butyryl-L-carnitine; C4.OH..C3.DC.: hydroxybutyryl-L-carnitine (malonyl-L-carnitine); C5.DC..C6.OH.: glutaryl-L-carnitine (hydroxyhexanoyl-L-carnitine); C5.OH..C3.DC.M.: hydroxyvaleryl-/-isovaleryl-/- methylbutyryl-L-carnitine (methylmalonyl-L-carnitine); C6:1: hexenoyl-L-carnitine; C7-DC: pimelyl-L-carnitine; C16-OH: hydroxyhexadecanoyl-L-carnitine; C16:2: hexadecadienyl-Lcarnitine; C18:1: octadecenoyl-L-carnitine; C18:1-OH: hydroxyoctadecenoyl-L-carnitine; C18:2: octadecadienyl-L-carnitine; FC: fold change; H1: hexose; lysoPC.a.: lysophosphatidylcholine acyl; n.d.: not determined; PC.aa.: phosphatidylcholine diacyl; PC.ae.: phosphatidylcholine acyl-alkyl; SM: sphingomyelin; SM..OH..: hydroxysphingomyeline. Click here for file
