# Supplementary material for: C57Bl/6 N mice on a western diet display reduced intestinal and hepatic cholesterol levels despite a plasma hypercholesterolemia
Source: BMC Genomics. 2012 Mar 6;13:84. doi: 10.1186/1471-2164-13-84 (PMC3319424; doi:10.1186/1471-2164-13-84)
Supplement: Additional file 1 — Table S1. Primer sequences. [file 1471-2164-13-84-S1.PDF]

Supplementary table 1. Primer sequences

Symbol	Gene name	Forward primer	Reverse primer
**Gapdh**	Glyceraldehyde-3-phosphate dehydrogenase	CCTGGAGAAACCTGCCAAGTATG	GAGTGGGAGTTGCTGTTGAAGTC
**Actb**	β-actin	CCACTGCCGCATCCTCTTCC	GCCACAGGATTCCATACCCAAGA
**Hprt**	Hypoxanthine guanine phophoribosyl transferase	GTCGTGATTAGCGATGATGAACC	GTCTTTCAGTCCTGTCCATAATCAG
**Srebp-2**	Sterol regulatory element binding factor 2	GGCACACTGCAGACCCTTGC	GAAGGGCGGCTGTCTGGATG
**Hmgcr**	3-hydroxy-3-methylglutaryl-Coenzyme A reductase	TTTCTAGAGCGAGTGCATTAGC	CAAGGCATTCCACAAGAGCGTC
**Pmvk**	Phosphomevalonate kinase	CTCTGGTCCACTCAAGGAGGAG	GCTGGGACACGCCTTCCAC
**Cyp51**	Cytochrome P450, family 51	CACGCTGCCTGGCTATTGC	ACGCCCGTCCTTGTATGTAGAATC
**Dhcr7**	7-dehydrocholesterol reductase	CTGCCCAACTGTATGCCTTGTG	CTTGCAGCCCATTCACCTCATAC
