# Supplementary material for: C57Bl/6 N mice on a western diet display reduced intestinal and hepatic cholesterol levels despite a plasma hypercholesterolemia
Source: BMC Genomics. 2012 Mar 6;13:84. doi: 10.1186/1471-2164-13-84 (PMC3319424; doi:10.1186/1471-2164-13-84)
Supplement: Additional file 2 — Table S2. Effect of a chronic Western diet on final body weight, cumulative food, energy, water, macronutrient and cholesterol intake and energy assimilation. [file 1471-2164-13-84-S2.PDF]

Supplementary table 2. Effect of a chronic high fat diet on final body weight, cumulative food, energy, water, macronutrient and cholesterol intake and energy assimilation [a]

	Control	Western diet	p-value
**Weight (g)**	29.7 ± 0.4	43.8 ± 1.1	< 0.001
**Food (g)**	307 ± 2	263 ± 2	< 0.001
**Energy (kcal)**	1318 ± 10	1580 ± 14	< 0.001
**Water (ml)**	329 ± 12	229 ± 8	< 0.001
**Protein (g)**	59.5 ± 0.5	42.4 ± 0.4	< 0.001
**Fat (g)**	28.5 ± 0.2	133.8 ± 1.2	< 0.001
**Carbohydrates (g)**	170.9 ± 1.3	46.8 ± 0.4	< 0.001
**Cholesterol (mg)**	0.0 ± 0.0	76.1 ± 0.7	< 0.001
**Digested Energy (kcal/d)**			
**Day 4 to 11**	15.9 ± 0.3	20.2 ± 1.5	0.047
**Day 46 to 53**	14.4 ± 0.4	18.0 ± 0.5	0.002
**Day 74 to 81**	15.7 ± 0.6	21.0 ± 0.4	< 0.001

[a] Results are expressed as mean ± SEM (n = 12). For digested energy (n = 5), data were analyzed using the mixed model. The effect of diet and time was significant.
