# Supplementary figures and images for: C57Bl/6 N mice on a western diet display reduced intestinal and hepatic cholesterol levels despite a plasma hypercholesterolemia
Source: BMC Genomics. 2012 Mar 6;13:84. doi: 10.1186/1471-2164-13-84 (PMC3319424; doi:10.1186/1471-2164-13-84)

Supplementary figure 1.

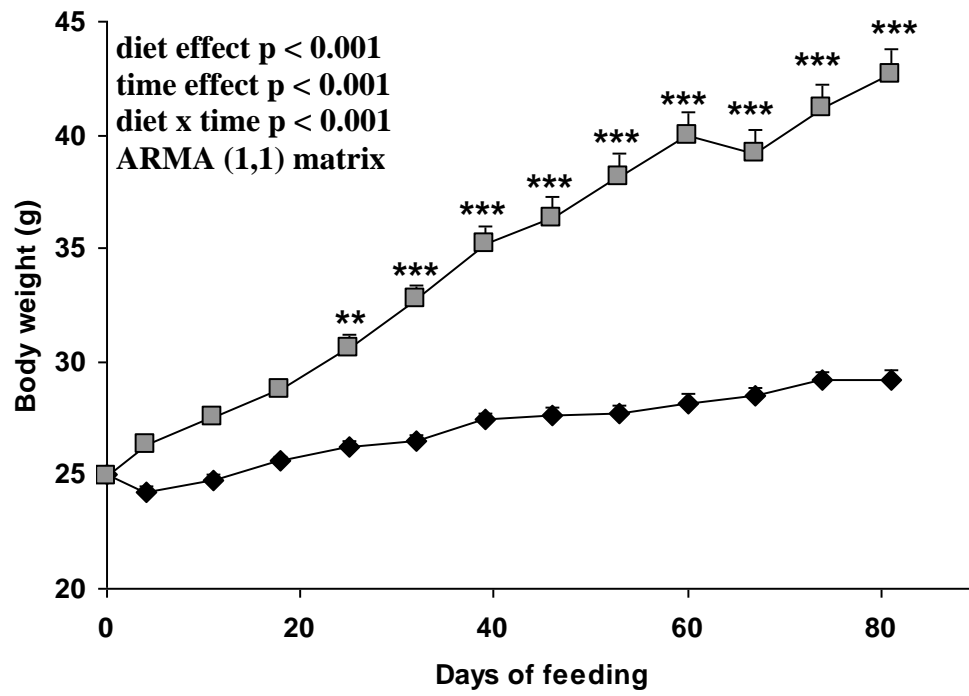

Supplement: Additional file 3 — Figure S1. Body weight development in mice fed the different diets. Symbols: black diamonds, control diet; grey squares, Western diet. Data are presented as mean ± SEM (n = 12). ** p < 0.01; *** p < 0.001. [file 1471-2164-13-84-S3.PDF]

Supplementary figure 2.

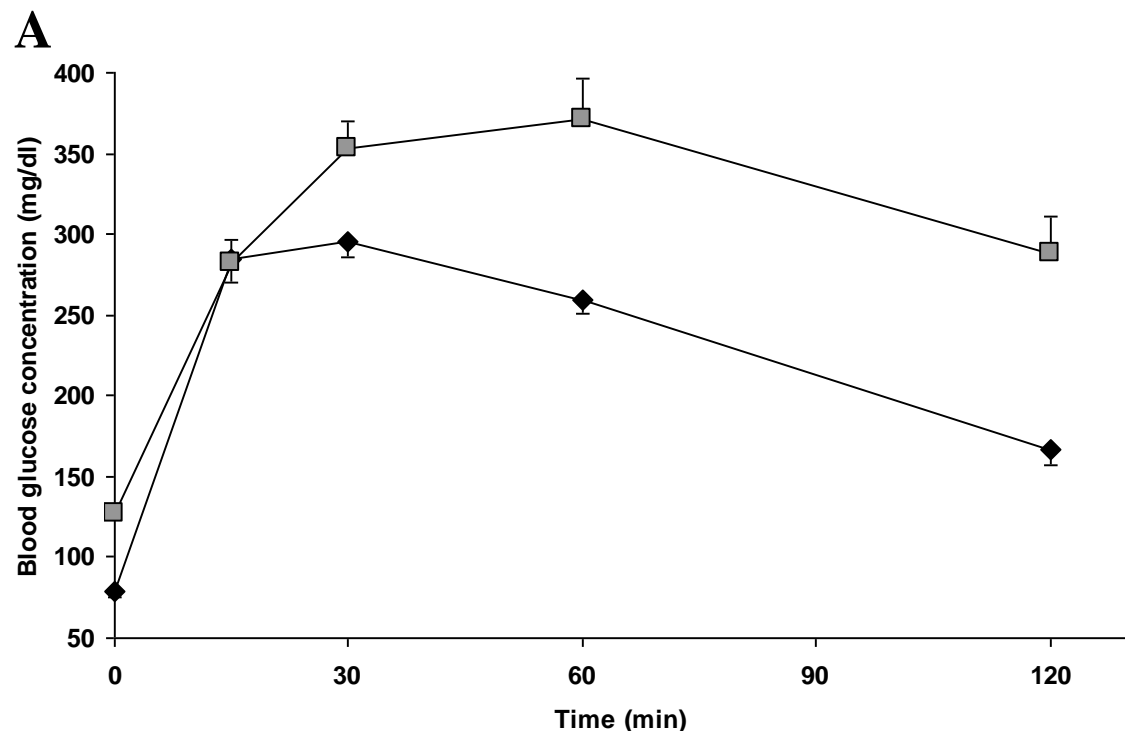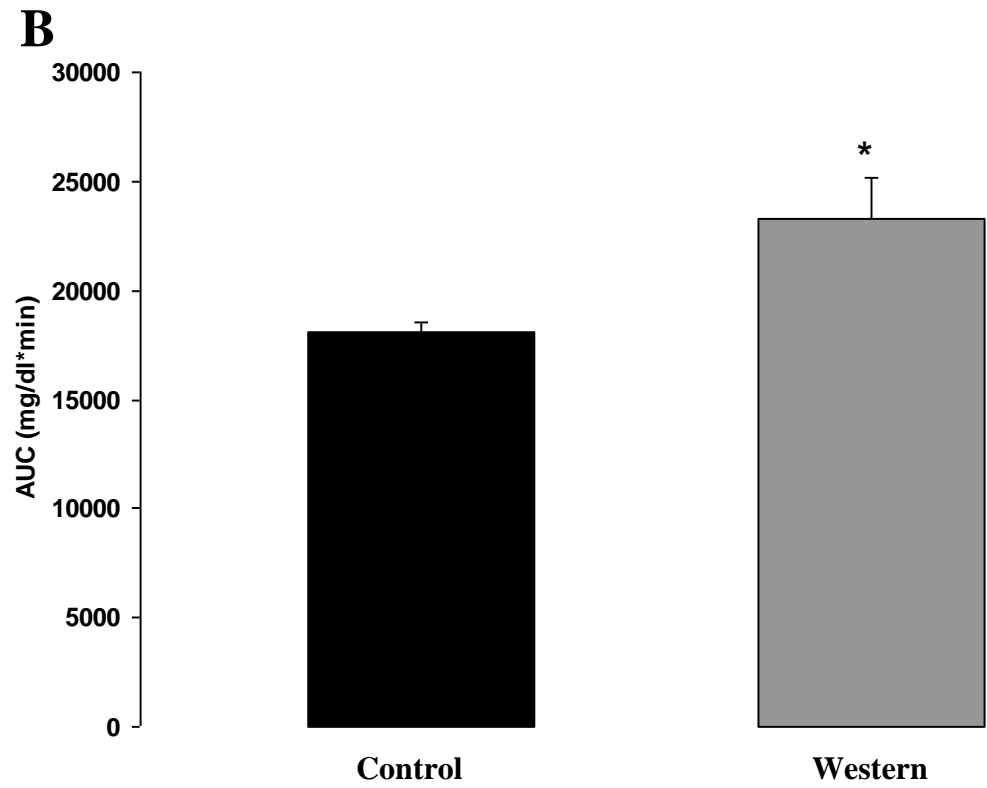

Supplement: Additional file 4 — Figure S2. Glucose tolerance in mice fed the different diets for 9 weeks. A: A glucose tolerance test was carried out after 9 weeks of dietary intervention following 14 h of food deprivation. Blood was collected from the tail vein 0, 15, 30, 60 and 120 min after an intraperitoneal 20% glucose solution injection (10 ml/kg of body weight) and blood glucose was measured. Symbols: black diamonds, control diet; grey squares, Western diet. B: Area Under the Curve (AUC) calculated from the glucose tolerance test. Black bar: control diet; grey bar: Western diet. Data are presented as mean ± SEM (n = 12). * p < 0.05. [file 1471-2164-13-84-S4.PDF]
