# Supplementary material for: C57Bl/6 N mice on a western diet display reduced intestinal and hepatic cholesterol levels despite a plasma hypercholesterolemia
Source: BMC Genomics. 2012 Mar 6;13:84. doi: 10.1186/1471-2164-13-84 (PMC3319424; doi:10.1186/1471-2164-13-84)
Supplement: Additional file 5 — Table S3. Effect of a chronic Western diet on selected blood parameters. [file 1471-2164-13-84-S5.PDF]

Supplementary table 3. Effect of a chronic high fat diet on selected blood parameters [a]

	Control	Western diet	p-value
**Glucose (mg/dl)**	213 ± 9	257 ± 9	0,002
**Insulin (ng/ml)**	0.49 ± 0.11	3.03 ± 0.52	< 0.001
**Cholesterol (mg/dl)**	95 ± 4	181 ± 5	< 0.001
**HDL cholesterol (mg/dl)**	71 ± 3	123 ± 3	< 0.001
**TG (mg/dl)**	79 ± 5	78 ± 4	0.862

[a] Results are expressed as mean ± SEM (n = 9 - 12).
